# Streptococcal toxic shock syndrome with acute respiratory distress syndrome following adenotonsillectomy in a child: a case report

**DOI:** 10.3389/fped.2025.1717143

**Published:** 2025-11-28

**Authors:** Wenhai Yang, Zhijun Lai, Henian Li, Keze Ma

**Affiliations:** Department of Pediatric Intensive Care Unit, Dongguan Children’s Hospital Affiliated to Guangdong Medical University, Dongguan, China

**Keywords:** *Streptococcus pyogenes*, streptococcal toxic shock syndrome (STSS), acute respiratory distress syndrome (ARDS), continuous renal replacement therapy (CRRT), adenotonsillectomy

## Abstract

This article reports a case of an 8-year-old child who developed Streptococcal Toxic Shock Syndrome (STSS) and Acute Respiratory Distress Syndrome (ARDS) following adenotonsillectomy, summarizing the diagnostic and treatment process. Preoperative blood tests, CRP levels, and chest x-rays of the child showed no abnormalities. However, within two hours after surgery, the patient exhibited high-grade fever, dyspnea, and altered mental status, rapidly progressing to shock and ARDS. Although shock was not diagnosed promptly in the early stage, leading to non-standardized fluid resuscitation therapy, blood pressure and heart rate eventually stabilized after adjusting the fluid resuscitation protocol and combining vasoactive medications. The diagnosis of STSS was ultimately confirmed based on sputum bacterial culture from endotracheal intubation and targeted next-generation sequencing(tNGS) results. STSS manifests with sudden onset and rapid progression clinically. It is often misdiagnosed in the early stages, resulting delayed treatment of shock and consequently an extremely high mortality rate.

## Background

*Streptococcus pyogenes*, also known as Group A *Streptococcus* (GAS), is one of the most common pathogens causing infections in children. Streptococcal toxic shock syndrome (STSS) is a severe manifestation of GAS infection, with mortality rates reported between 23% and 81% (1). STSS is associated with Group A streptococcal infections triggered by factors such as viral infections (e.g., influenza), pharyngitis, and local soft tissue trauma. Cases of STSS following otorhinolaryngological surgeries have also been reported. A documented case of STSS following otorhinolaryngological surgery was reported in 2003 (2). Affected children may present with neurological symptoms such as confusion, delirium, and coma. The organ dysfunction and shock observed in STSS resemble those of septic shock, but renal impairment/failure may occur earlier (3).

## Case description

**Figure 1:**
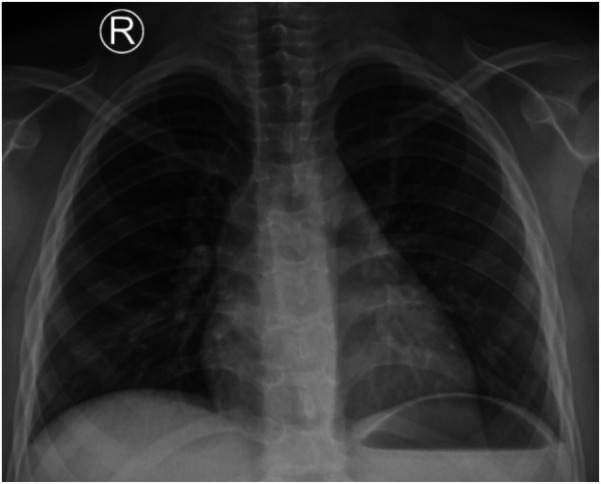
Chest x-ray of the child on the day of admission.

A female child, 8 years old, was admitted to the hospital due to “concerns for obstructive sleep apnea” Preoperative blood tests, CRP, and chest x-ray showed no abnormalities (Figure 1). On the second day of admission, she underwent bilateral tonsillectomy and adenoid ablation via nasal endoscopy under general anesthesia using low-temperature plasma. Approximately 2 h after surgery, she developed dyspnea, respiratory distress, and drowsiness, and was transferred to the Pediatric Intensive Care Unit (PICU).

Upon admission to PICU, physical examination revealed: body temperature 38 °C, pulse 132 beats/min, respiratory rate 42 breaths/min, blood pressure 129/97 mmHg (1 mmHg = 0.133 kPa). Glasgow Coma Scale (GCS) score was 12 (E4V3M5). The child presented with cyanotic lips, tachypnea, diffuse erythema of the skin, and marked inspiratory retractions. Bilateral coarse breath sounds with moist rales were audible on pulmonary auscultation. Cardiac examination showed a regular rhythm without audible murmurs across all valve areas.

Following PICU admission, the initial arterial blood gas analysis (on 36% FiO_2_) revealed pH 7.26, PaCO_2_ 53.9 mmHg, PaO_2_ 51.1 mmHg, and lactate 2.5 mmol/L, consistent with type II respiratory failure. Endotracheal intubation and mechanical ventilation were promptly initiated. Empirical antibiotic therapy with piperacillin-tazobactam was commenced, along with active temperature control, analgesia, and sedation.

8 h after surgery, the child's blood pressure dropped to 86/42 mmHg with oliguria. Rapid fluid resuscitation was initiated (5 mL/kg·h). By 11 h postoperatively, her blood pressure was 82/40 mmHg with anuria. Total fluid intake over 3 h was 15 mL/kg. Repeat blood gas analysis showed lactate level of 6.5 mmol/L, and norepinephrine was started at 0.2 μg/(kg·min) for vasopressor support.

By 14 h postoperatively,the patient remained hypotensive (82/33 mmHg) and tachycardic (140 beats/min), with persistent anuria. Total fluid intake over 6 h was 1,100 mL. Repeat blood gas analysis showed lactate level of 7.2 mmol/L. Norepinephrine was increased to 0.4 μg/(kg·min). Bedside echocardiography indicated a decreased ejection fraction (EF) of 36%, so dobutamine was added at 10 μg/(kg·min) for inotropic support. The central venous pressure (CVP) was 6 cmH_2_O.Non-invasive hemodynamic monitoring showed: cardiac output (CO) 9.1 L/min (normal range 4.0–7.5 L/min), stroke volume variation (SVV) 18% (normal 5%–15%), systemic vascular resistance (SVR) 548 dyn·s/cm^5^ (normal 697–1,294 dyn·s/cm^5^), and index of contractility (ICON) 118.9 (normal 49–91). The elevated SVV and low SVR suggested hypovolemic, low cardiac output, and low vascular resistance shock.

Considering the child's weight was over 50 kg, volume expansion was initiated (total fluid volume 54 mL/kg over 3 h after starting expansion), while continuing dobutamine and norepinephrine. By the 15th hour, her blood pressure gradually increased to 115/72 mmHg. CVP increased to 14 cmH_2_O. The repeated blood gas analysis revealed a progressive elevation in lactate to 9.2 mmol/L. Due to persistent anuria for over 6 h, continuous renal replacement therapy (CRRT) was initiated using citrate anticoagulation.

By 16 h postoperatively, her blood pressure was 104/60 mmHg, urine output increased to 2.31 mL/kg, and lactate decreased to 7.2 mmol/L. By the 17th postoperative hour, blood pressure was 110/70 mmHg, urine output 7.69 mL/kg, and lactate further decreased to 6.7 mmol/L. By the 20th postoperative hour, blood pressure was 115/59 mmHg, urine output 3.85 mL/kg, but lactate increased again to 9.3 mmol/L.

Vital signs, urine output, and lactate levels during CRRT are detailed in Tables 1, 2. After excluding hypoxemia and hypoperfusion as causes of lactate elevation, it was attributed to citrate anticoagulation used during CRRT. CRRT was discontinued after approximately 7 h. Following its cessation, lactate levels showed a gradual declining trend. Urine output and lactate changes are detailed in Table 3.

**Table 1:** Heart rate and blood pressure of the child during CRRT.

Item	Hour 15	Hour 16	Hour 17	Hour 18	Hour 19	Hour 20	Hour 21
Heart Rate(beats/min)	170	168	169	140	145	144	137
Systolic Blood Pressure (mmHg)	115	104	110	120	122	115	130
Diastolic Blood Pressure (mmHg)	72	60	70	56	50	59	56

**Table 2:** Urine output and lactate levels of the child during CRRT.

Item	Hour 15	Hour 16	Hour 17	Hour 18	Hour 19	Hour 20	Hour 21
Urine Output [ml/(kg·h)]	0.38	2.31	7.69	4.23	5.19	3.85	3.85
Lactate (mmol/L)	9.2	7.2	6.7	/	/	9.3	/

**Table 3:** Urine output and lactate levels of the child after CRRT discontinuation.

Item	Day 3	Day 4	Day 5	Day 6	Day 7	Day 9	Day 12	Day 13
Lactate (mmol/L)	7.0	4.4	4.1	1.9	1.2	1.1	1.4	1.3
Urine Output [mL/(kg·h)]	2.95	2.09	1.74	0.88	3.46	2.67	1.68	2.17

On Day 3 of hospitalization, the child's blood pressure and urine output remained relatively stable, permitting a gradual weaning of vasoactive medications. However, a decrease in oxygen saturation was observed, accompanied by the appearance of copious thin, pale-red secretions in the endotracheal tube. A repeat chest x-ray revealed diffuse bilateral pulmonary infiltrates. Ventilator settings were increased, and after initiating prone positioning therapy, oxygen saturation gradually improved, fluctuating between 93% and 95%.

On Day 4 of hospitalization, pathogen test results returned: both sputum culture and tNGS detected *Streptococcus pyogenes*（Both collected immediately post-intubation and sent for analysis）. Antimicrobial susceptibility testing indicated sensitivity to penicillin and resistance to clindamycin. According to the Chinese Expert Consensus on Diagnosis, Treatment, and Prevention of Group A Streptococcal Infections in Children (2022) (4), combination therapy with clindamycin is recommended for inhibiting toxin and superantigen production by S. pyogenes. Accordingly, the antibiotic regimen was adjusted to penicillin combined with clindamycin.

**Figure 2:**
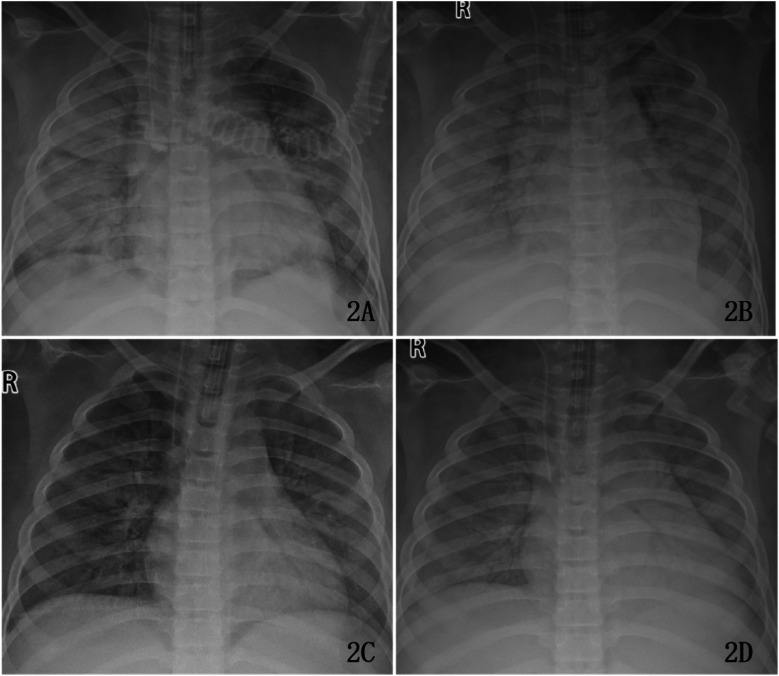
Changes in pulmonary infiltrates during ARDS treatment in the child. **(A)** Chest x-ray on day 3; **(B)** Chest x-ray on day 5; **(C)** Chest x-ray on day 6; **(D)** Chest x-ray on day 14.

**Figure 3:**
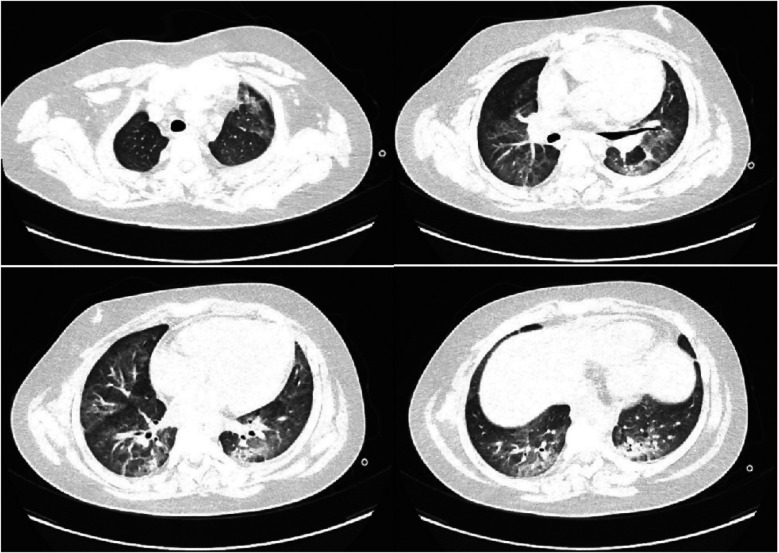
Chest CT findings of the child on day 28 of hospitalization.

Following the adjustment in treatment, the infection was gradually brought under control, and pulmonary infiltrates showed progressive resolution (Figure 2). Subsequent follow-up tests showed a steady decline in infection markers, and arterial blood gas analysis indicated gradual improvement in ventilation and oxygenation function.Ventilator settings were gradually weaned.The endotracheal tube was removed on Day 15, and the child was switched to non-invasive ventilation support. Non-invasive ventilation was discontinued on Day 19. The patient was transferred to the general ward on Day 22. A CT scan on Day 28 showed uneven aeration in parts of the lung parenchyma and focal consolidations/atelectasis in bilateral dorsal segments (Figure 3). After a total hospitalization of 41 days, the child was discharged fully recovered.

## Discussion

STSS must be differentiated from staphylococcal toxic shock syndrome. The latter is less commonly associated with soft tissue infections and localized pain, but more frequently presents with symptoms such as skin rash, vomiting, and diarrhea (5). The child in this case exhibited high fever, diffuse erythema of the trunk, and signs of circulatory dysfunction. The severity of circulatory dysfunction, assessed using the Phoenix Sepsis Score (PSS), was 8 points (respiratory system score 2, cardiovascular system score 6), meeting the diagnostic criteria for septic shock. Combined with the detection of Streptococcus pyogenes in both sputum culture and sputum tNGS, a diagnosis of STSS was established.

STSS progresses with extreme rapidity and can advance to multiple organ failure within a short period. Early recognition and aggressive treatment of shock is critical. A 2016 study indicated that nearly all patients with STSS required both endotracheal intubation and renal replacement therapy (RRT) (6). However, with increased global emphasis on Streptococcus pyogenes infections in recent years and optimized treatment approaches including antimicrobial therapy and intravenous immunoglobulin (IVIG), the necessity for such interventions has shown a declining trend. Previous reports have documented two pediatric cases of STSS following tonsillectomy in which patients exhibited signs of infection prior to surgery, with both cases demonstrating rapid clinical deterioration shortly after the procedure (2). In contrast, the present case showed no preoperative evidence of infection; however, the patient developed fever within 2 h postoperatively, rapidly progressing to shock and multiple organ failure. The absence of active infection preoperatively was supported by unremarkable CRP and interleukin-6 levels upon admission, as well as normal chest x-ray findings. This clinical course suggests that the STSS in this case likely resulted from invasive infection by locally colonized Group A Streptococcus in the postoperative period. Approximately 8% of healthy children are reported to be asymptomatic carriers of GAS (7), which may lead to infection under conditions such as viral infection or impaired local mucosal barrier function.

Reviewing this case, it is postulated that following tonsillectomy and adenoid ablation, partial reopening of the nasopharyngeal passage led to the reflux of accumulated secretions from the sinuses and nasal cavity. In the postoperative state, with reduced airway protective reflexes due to lingering anesthesia, aspiration of secretions likely caused postoperative pulmonary infection. Concurrently, the disruption of the mucosal barrier at the surgical site provided an opportunity for invasive GAS infection to establish and progress.

Since no signs of active infection was detected preoperatively, and symptoms such as high fever and tachypnea emerged extremely shortly after surgery, clinicians initially underestimated the possibility of secondary infection and misjudged the potential severity of subsequent deterioration. This led to a delay in recognizing and managing septic shock. Furthermore, the child weighed 54 kg with a height of 140 cm. Adhering strictly to pediatric septic shock fluid resuscitation guidelines risked fluid overload and right heart dysfunction. Therefore, subsequent fluid resuscitation was guided by adult septic shock protocols (total fluid ≥30 mL/kg in the first 3 h) (8). After adequate fluid resuscitation, the child's blood pressure recovered, and lactate levels decreased.

The population in PICU encompasses a wide range of ages, weights, and body sizes. Even for the same condition, treatment plans must be individualized, choosing between adult or pediatric consensus guidelines based on the child's age, weight, and other factors.

The management of STSS largely aligns with the principles for septic shock. Antimicrobial therapy is foundational, and there is broad consensus domestically and internationally on using a combination of clindamycin and a β-lactam antibiotic (7, 9, 10). Although the GAS strain isolated from the deep sputum culture in this case was resistant to clindamycin, clindamycin inhibits superantigen production and reduces toxin release by GAS through a mechanism independent of its antibacterial activity (11). Therefore, the susceptibility of the GAS strain to clindamycin does not affect these beneficial effects. For patients receiving combination therapy with clindamycin, it should be discontinued after normalization of body temperature, improvement of systemic symptoms, and resolution of shock (12).

CRRT is important for organ support in children with STSS. However, during CRRT using citrate anticoagulation in this case, a paradoxical rise in lactate was observed. This elevation occurred despite adequate oxygen saturation, stabilized blood pressure, and improved urine output, thereby excluding hypoxia or hypoperfusion as the primary causes. Consequently, iatrogenic factors were strongly considered for the rising lactate. During regional citrate anticoagulation, approximately 50% of the calcium chelated by citrate is cleared by the filter (11), while the remainder enters the systemic circulation. Citrate is metabolized via the tricarboxylic acid (TCA) cycle in mitochondria. In shock states, mitochondrial function is impaired. The influx of citrate adds an additional burden to the already compromised mitochondria, preventing pyruvate from entering the TCA cycle normally (13). This leads to accumulation of pyruvate in the cytoplasm and its subsequent conversion to lactate. After discontinuing CRRT, the child's lactate levels gradually decreased.

## Conclusion

STSS triggered by group A streptococcal infection following adenotonsillectomy is a rare etiology. For pediatric patients presenting with fever and rapid clinical deterioration, clinicians should maintain a high index of suspicion for possible STSS. Given the rapid progression of STSS, early recognition and standardized management could potentially prevent disease progression and subsequent complications in such pediatric cases.

## Data Availability

The datasets presented in this study can be found in online repositories. The names of the repository/repositories and accession number(s) can be found in the article/Supplementary Material.
